# Dietary Nickel Chloride Induces Oxidative Intestinal Damage in Broilers

**DOI:** 10.3390/ijerph10062109

**Published:** 2013-05-23

**Authors:** Bangyuan Wu, Hengmin Cui, Xi Peng, Jing Fang, Zhicai Zuo, Junliang Deng, Jianying Huang

**Affiliations:** Key Laboratory of Animal Diseases and Environmental Hazards of Sichuan Province, College of Veterinary Medicine, Sichuan Agricultural University, Ya’an 625014, China; E-Mails: wubangyuan2008@163.com (B.W.); pengxi197313@163.com (X.P.); fangjing4109@163.com (J.F.); zzcjl@126.com (Z.Z.); dengjl213@126.com (J.D.); hjy19860316@163.com (J.H.)

**Keywords:** broiler, intestinal mucosa, nickel chloride, oxidative damage

## Abstract

The purpose of this study was to investigate the oxidative damage induced by dietary nickel chloride (NiCl_2_) in the intestinal mucosa of different parts of the intestine of broilers, including duodenum, jejunum and ileum. A total of 240 one-day-old broilers were divided into four groups and fed on a corn-soybean basal diet as control diet or the same basal diet supplemented with 300, 600 or 900 mg/kg NiCl_2_ during a 42-day experimental period. The results showed that the activities of superoxide dismutase (SOD), catalase (CAT) and glutathione peroxidase (GSH-Px), and the ability to inhibit hydroxy radical and glutathione (GSH) content were significantly (*p* < 0.05 or *p* < 0.01) decreased in the 300, 600 and 900 mg/kg groups in comparison with those of the control group. In contrast, malondialdehyde (MDA) content was significantly (*p* < 0.05 or *p* < 0.01) higher in the 300, 600 and 900 mg/kg groups than that in the control group. It was concluded that dietary NiCl_2_ in excess of 300 mg/kg could cause oxidative damage in the intestinal mucosa in broilers, which finally impaired the intestinal functions including absorptive function and mucosal immune function. The oxidative damage might be a main mechanism on the effects of NiCl_2_ on the intestinal health of broilers.

## 1. Introduction

Nickel (Ni) is a naturally occurring metal element of widespread distribution in the environment and with many industrial and commercial uses. Ni is also a nutritionally essential trace element for many animal species, micro-organisms and plants, and therefore either deficiency or toxicity symptoms can occur when too little or too much Ni is taken up, respectively [1,2,3,4]. Ni forms stable stoichiometric complexes with amino acids [5,6,7], proteins [8] and other organic compounds [9]. Two biological compounds have been found to essentially contain Ni: urease from jack beans [10,11,12] and carbon monoxide dehydrogenase from clostridia [13,14,15].

In 1990 the International Agency for Research on Cancer (IARC) classified Ni compounds as carcinogenic to humans [16]. The adverse effects of Ni on animals following acute, subchronic, and chronic exposure periods can be classified according to their systemic, immunologic, neurologic, reproductive, or developmental aspects [17]. 

The findings with chicks suggested that Ni may play a role in the function of membranes [18]. Besides the function of nutritional absorption, the intestinal mucosa displays complex defense mechanisms [19]. In relation to prooxidant-antioxidant balance, the intestinal mucosa occupies a unique position [20]. The detection and measurement of lipid peroxidation is most frequently cited to support the involvement of free-radical reactions in toxicology and disease [21]. The antioxidant capacity of the intestinal mucosa is crucial for achieving healthy intestinal function in broilers. As a nutritionally essential trace element, Ni-induced oxidative damage in the intestines of animals has not been reported to date. Ni exposure causes formation of free radicals in various tissues in both human and animals, and enhances lipid peroxidation. Free radical generation from the reaction of Ni-thiol complexes and molecular oxygen, and/or lipid hydroperoxides, can play an important role in the mechanism(s) of Ni toxicity [22]. It has been reported that changes of superoxide dismutase (SOD), catalase (CAT), glutathione peroxidase (GSH-Px) and glutathione reductase (GSSG-R) activity, and glutathione (GSH) and malondialdehyde (MDA) contents induced by Ni are observed in the blood, liver or kidney of rats [23]. However, at present there are no reports about the impact of nickel chloride (NiCl_2_) on the oxidative damage in the intestines (duodenum, jejunum and ileum) of animals. Parameters that were used to represent the oxidative damage in this study included the activities of SOD, CAT and GSH-Px, and ability to inhibit hydroxy radical, and contents of GSH and MDA. The aims were to provide new evidence for further understanding the mechanism about the effects of NiCl_2_ on intestinal health. 

## 2. Materials and Methods

### 2.1. Chickens and Diets

240 one-day-old healthy avian broilers were randomly divided by body weight into four groups with 60 broilers in each group. Broilers were housed in cages with electrical heaters and were provided with feed and water as well as undermentioned experimental diets *ad libitum* for 42 days. A corn-soybean basal diet formulated by the National Research Council (NRC) [24] was the control diet. NiCl_2_ was mixed into the corn-soybean basal diet to produce the experimental diets containing 300, 600 and 900 mg/kg NiCl_2_, respectively. 

### 2.2. Detection of Oxidative Damage Parameters in the Intestinal Mucosa

At 14, 28, and 42 days of age during the experiment, five broilers in each group were humanely killed and the intestinal tract were immediately removed and chilled to 0 °C in 0.85% sodium chloride (NaCl) solution, and divided into the duodenum, jejunum and ileum. A segment of approximately 4 cm in length was collected from the middle section of each intestinal region, and then dissected and thoroughly cleaned with 0.85% NaCl solution. The mucosa was carefully scraped from the luminal face of the taken intestinal segments with clean slides and put into 2.5 mL aseptic tubes and then stored at −80 °C prior to the measurement of oxidative damage parameters. For detection, the scraped mucosa was weighed and homogenized in nine volumes of ice-cold 85% NaCl solution in a chilled homogenizer, and immediately centrifuged at 3,000 × g for 10 min at 4 °C. The pellet was discarded and the supernatant was conserved for future analysis [25].

After determining the amount of total protein (A045-2, LOT: 201211) in the supernatant of the mucosa homogenate by the Bradford method [26], the activities of SOD, CAT and GSH-Px, and ability to inhibit hydroxy radical, and contents of MDA and GSH in the supernatant were detected by biochemical methods following the instructions of the corresponding reagent kits (SOD: Cat. No.: A001-1, LOT: 201211; CAT: Cat. No.: A007, LOT: 201211; GSH-Px: Cat. No.: A005, LOT: 201211; ability to inhibit hydroxy radical: Cat. No.: A018, LOT: 201211; MDA: Cat. No.: A003-2, LOT: 201211; GSH: Cat. No.: A006, LOT: 201211, all purchased from Nanjing Jiancheng Bioengineering Institute of China, Nanjing, China). The absorbance of SOD, CAT, GSH-Px, ability to inhibit hydroxy radical, MDA, GSH and total protein were measured at 550, 240, 412, 550, 532, 420 nm and 590 nm, respectively, with a Varioskan Flash microtiter plate reader (Thermo, MA, USA). 

### 2.3. Statistical Analysis

 The significance of difference among four groups was analyzed by variance analysis, and results presented as means ± standard deviation (*X* ± S). The analysis was performed under SPSS 12.0 for windows. A value of *p* < 0.05 was considered significant. 

## 3. Results

### 3.1. Changes of the SOD Activities

The SOD activities of duodenum were lower (*p* < 0.05) in the 300 mg/kg group at 42 days of age and were significantly (*p* < 0.05 or *p* < 0.01) lower in the 600 and the 900 mg/kg groups than those in the control group from 14 to 42 days of age. The SOD activities of jejunum and ileum were significantly (*p* < 0.05 or *p* < 0.01) decreased in the 300, 600 and 900 mg/kg groups in comparison with those of control group from 14 to 42 days of age (Table 1).

**Table 1:** Change of the T-SOD activity (U/mgprot) in the intestinal mucosa.

Days	Groups	Duodenum	Jejunum	Ileum
14 Days	Control group	78.61 ± 3.37	68.77 ± 2.86	63.60 ± 2.73
	300 mg/kg group	76.91 ± 3.60	65.60 ± 2.30	62.90 ± 2.38
	600 mg/kg group	73.38 ± 4.34	67.45 ± 2.80	61.72 ± 2.72
	900 mg/kg group	72.16 ± 2.67 *	63.97 ± 5.51	59.52 ± 2.14 *
28 Days	Control group	76.18 ± 4.69	67.00 ± 4.37	67.76 ± 3.71
	300 mg/kg group	72.10 ± 5.06	61.66 ± 3.19 *	62.80 ± 2.85 *
	600 mg/kg group	63.24 ± 5.89 **	59.33 ± 4.56 **	60.62 ± 3.82 **
	900 mg/kg group	63.24 ± 5.89 **	52.50 ± 4.31 **	59.60 ± 2.05 **
42 Days	Control group	76.75 ± 4.06	66.94 ± 3.49	70.90 ± 4.56
	300 mg/kg group	69.07 ± 2.29 *	60.65 ± 4.63 *	62.73 ± 3.38 **
	600 mg/kg group	63.29 ± 3.28 **	58.63 ± 3.23 **	61.47 ± 3.08 **
	900 mg/kg group	64.47 ± 6.84 **	52.32 ± 3.20 **	56.67 ± 3.75 **

Data are presented with the means ± standard deviation (n = 5); * *p* < 0.05, compared with the control group, ** *p* < 0.01, compared with the control group; Data were analyzed by variance analysis using SPSS 16.0 software.

### 3.2. Changes of the CAT Activities

As showed in Table 2, the CAT activities of duodenum, jejunum and ileum were significantly (*p* < 0.05 or *p* < 0.01) reduced in the 300, 600 and 900 mg/kg groups when compared with those of the control group from 14 to 42 days of age. 

**Table 2:** Change of the CAT activity (U/gprot) in the intestinal mucosa.

Days	Groups	Duodenum	Jejunum	Ileum
14 Days	Control group	494.59 ± 14.43	467.09 ± 17.61	354.34 ± 15.94
	300 mg/kg group	492.29 ± 18.40	459.79 ± 15.82	350.79 ± 25.61
	600 mg/kg group	484.78 ± 23.97	457.28 ± 16.31 *	346.78 ± 16.66
	900 mg/kg group	481.46 ± 17.76 *	454.79 ± 14.18 *	343.54 ± 23.89 *
28 Days	Control group	479.96 ± 10.82	459.96 ± 10.82	348.71 ± 18.33
	300 mg/kg group	468.36 ± 23.60 *	448.36±13.60*	337.61 ± 15.36 *
	600 mg/kg group	467.73 ± 17.34 *	447.73 ± 27.34 *	334.73 ± 9.08 *
	900 mg/kg group	460.43 ± 14.50 **	440.43 ± 14.50 **	330.43 ± 14.50 **
42 Days	Control group	470.29 ± 10.04	455.29 ± 15.20	345.79 ± 16.43
	300 mg/kg group	454.18 ± 14.32 **	441.68 ± 13.75 **	331.18 ± 24.27 **
	600 mg/kg group	452.56 ± 23.24 **	437.56 ± 18.80 **	326.06 ± 19.22 **
	900 mg/kg group	445.75 ± 15.68 **	428.25 ± 22.79 **	317.75 ± 13.53 **

Data are presented with the means ± standard deviation (n = 5); * *p* < 0.05, compared with the control group, ** *p* < 0.01, compared with the control group; Data were analyzed by variance analysis using SPSS 16.0 software.

### 3.3. Changes of the GSH-Px Activities

The duodenal and ileac GSH-Px activities were significantly (*p* < 0.05 or *p* < 0.01) decreased in the 300, 600 and 900 mg/kg groups in comparison with those of control group from 14 to 42 days of age. The GSH-Px activities of jejunum were lower (*p* < 0.05) in the 300 mg/kg group at 42 days and were significantly (*p* < 0.05 or *p* < 0.01) lower in the 600 and the 900 mg/kg groups than those in the control group from 28 to 42 days of age (Table 3). 

**Table 3:** Change of the GSH-Px activity (U/mgprot) in the intestinal mucosa.

Days	Groups	Duodenum	Jejunum	Ileum
14 Days	Control group	427.83 ± 26.22	453.03 ± 50.07	330.44 ± 11.33
	300 mg/kg group	403.37 ± 24.81	419.59 ± 34.27	301.11 ± 41.15
	600 mg/kg group	372.82 ± 41.02 *	410.53 ± 27.16	289.89 ± 39.75
	900 mg/kg group	367.35 ± 34.10 *	404.86 ± 48.18	271.08 ± 36.61 *
28 Days	Control group	485.80 ± 24.83	422.94 ± 29.34	351.25 ± 26.56
	300 mg/kg group	432.76 ± 28.56 *	408.76 ± 39.64	311.25 ± 12.57 *
	600 mg/kg group	387.15 ± 38.39 **	367.17 ± 22.39 *	302.67 ± 21.87 *
	900 mg/kg group	355.06 ± 39.78 **	369.78 ± 26.99 *	257.06 ± 27.08 **
42 Days	Control group	431.78 ± 31.36	394.57 ± 15.91	351.91 ± 14.85
	300 mg/kg group	378.48 ± 11.26 **	340.17 ± 32.96 *	323.75 ± 4.59 **
	600 mg/kg group	358.28 ± 12.68 **	354.04 ± 23.11 *	315.49 ± 17.34 **
	900 mg/kg group	340.31 ± 15.21 **	336.79 ± 30.07 **	300.50 ± 11.05 **

Data are presented with the means ± standard deviation (n = 5); * *p* < 0.05, compared with the control group, ** *p* < 0.01, compared with the control group; Data were analyzed by variance analysis using SPSS 16.0 software.

### 3.4. Changes of the Abilities to Inhibit Hydroxy Radical

The abilities to inhibit hydroxy radical of duodenum and jejunum were significantly (*p* < 0.05 or *p* < 0.01) lower in the 300, 600 and 900 mg/kg groups than those in the control group from 14 to 42 days of age. 

**Table 4:** Change of the ability to inhibit hydroxy radical (U/mgprot) in the intestinal mucosa.

Days	Groups	Duodenum	Jejunum	Ileum
14 Days	Control group	192.48 ± 8.39	207.30 ± 13.79	192.97 ± 9.36
	300 mg/kg group	181.27 ± 7.41	191.63 ± 9.46	184.44 ± 10.46
	600 mg/kg group	178.50 ± 8.56 *	187.36 ± 12.34 *	189.46 ± 6.35
	900 mg/kg group	175.76 ± 7.44 *	181.57 ± 9.75 **	175.28 ± 8.72 *
28 Days	Control group	197.38 ± 11.73	210.22 ± 13.95	197.81 ± 8.70
	300 mg/kg group	189.57 ± 2.21 *	192.58 ± 8.20 *	179.18± 10.47 *
	600 mg/kg group	179.43 ± 8.32 **	184.32 ± 8.53 **	172.11 ± 10.59 **
	900 mg/kg group	180.07 ± 6.65 **	173.95 ± 6.45 **	164.77 ± 7.68 **
42 Days	Control group	217.46 ± 4.83	208.55 ± 13.49	195.62 ± 9.49
	300 mg/kg group	206.67 ± 8.83 *	181.51 ± 6.52 **	179.13 ± 5.47 **
	600 mg/kg group	202.10 ± 6.21 **	169.98 ± 14.29 **	175.82 ± 9.69 **
	900 mg/kg group	198.45 ± 6.41 **	175.86 ± 10.98 **	170.76 ± 4.30 **

Data are presented with the means ± standard deviation (n = 5); * *p* < 0.05, compared with the control group, ** *p* < 0.01, compared with the control group; Data were analyzed by variance analysis using SPSS 16.0 software.

The ability to inhibit hydroxy radical of ileum was significantly (*p* < 0.05 or *p* < 0.01) decreased in the 300, 600 and 900 mg/kg groups from 28 to 42 days of age, and were decreased (*p* < 0.05) in the 900 mg/kg group at 14 days of age, as shown in Table 4.

### 3.5. Changes of the GSH Contents

The GSH contents of duodenum and jejunum were markedly (*p* < 0.05 or *p* < 0.01) lower in the 300, 600 and 900 mg/kg groups than those in the control group from 14 to 42 days of age. The ileac GSH content was reduced (*p* < 0.05) in the 300 mg/kg group and significantly (*p* < 0.05 or *p* < 0.01) reduced in the 600 and 900 mg/kg groups when compared with that of the control group from 14 to 42 days of age (Table 5).

**Table 5:** Change of the GSH content (mg/gprot) in the intestinal mucosa.

Days	Groups	Duodenum	Jejunum	Ileum
14.Days	Control group	7.06 ± 0.75	5.47 ± 0.47	2.66 ± 0.31
	300 mg/kg group	6.84 ± 0.56	5.07 ± 0.46	2.30 ± 0.16
	600 mg/kg group	7.07 ± 0.35	4.43 ± 0.28 **	2.74 ± 0.55
	900 mg/kg group	6.10 ± 0.49 *	4.10 ± 0.30 **	2.08 ± 0.16 *
28 Days	Control group	7.06 ± 0.75	4.57 ± 0.23	2.60 ± 0.25
	300 mg/kg group	6.24 ± 0.34 *	3.69 ± 0.38 **	1.89 ± 0.46 *
	600 mg/kg group	6.06 ± 0.59 **	3.55 ± 0.36 **	1.95 ± 0.14 **
	900 mg/kg group	5.47 ± 0.20 **	2.96 ± 0.17 **	1.63± 0.41 **
42 Days	Control group	6.35 ± 0.37	4.57 ± 0.41	3.15 ± 0.21
	300 mg/kg group	5.48 ± 0.43 **	3.58 ± 0.25 **	2.58 ± 0.40 *
	600 mg/kg group	5.68 ± 0.28 *	3.25 ± 0.34 **	2.38 ± 0.17 **
	900 mg/kg group	4.92±0.32 **	3.45±0.41 **	1.95 ± 0.40 **

Data are presented with the means ± standard deviation (n = 5); * *p* < 0.05, compared with the control group, ** *p* < 0.01, compared with the control group; Data were analyzed by variance analysis using SPSS 16.0 software.

### 3.6. Changes of the MDA Contents

The MDA contents of duodenum and jejunum were increased (*p* < 0.05) in the 300 mg/kg group and markedly (*p* < 0.05 or *p* < 0.01) increased in the 600 and 900 mg/kg groups in comparison with those of the control group from 14 to 42 days of age. The ileac MDA content was higher (*p* < 0.05 or *p* < 0.01) in the 300, 600 and 900 mg/kg groups than that in the control group from 14 to 42 days of age (Table 6).

**Table 6:** Change of the MDA content (nmol/mgprot) in the intestinal mucosa.

Days	Groups	Duodenum	Jejunum	Ileum
14 Days	Control group	2.32 ± 0.04	2.20 ± 0.09	2.67 ± 0.33
	300 mg/kg group	2.47 ± 0.09	2.37 ± 0.14	3.06 ± 0.18
	600 mg/kg group	2.42 ± 0.25	2.48 ± 0.30	3.04 ± 0.19
	900 mg/kg group	2.61 ± 0.20 *	2.56 ± 0.20 *	2.97 ± 0.29
28 Days	Control group	1.99 ± 0.39	2.30 ± 0.15	2.07 ± 0.29
	300 mg/kg group	2.65 ± 0.22 *	2.93 ± 0.23 *	2.66 ± 0.33 *
	600 mg/kg group	2.79 ± 0.38 **	3.13 ± 0.49 **	3.09 ± 0.55 **
	900 mg/kg group	3.03 ± 0.26 **	3.56 ± 0.47 **	3.30 ± 0.17 **
42 Days	Control group	2.01 ± 0.48	2.66 ± 0.27	2.62 ± 0.35
	300 mg/kg group	2.79 ± 0.42 *	3.29 ± 0.30 *	3.46 ± 0.59 *
	600 mg/kg group	3.27 ± 0.39 **	3.82 ± 0.48 **	3.50 ± 0.22 *
	900 mg/kg group	3.41 ± 0.46 **	3.65 ± 0.49 **	4.12 ± 0.60 **

Data are presented with the means ± standard deviation (n = 5); * *p* < 0.05, compared with the control group, ** *p* < 0.01, compared with the control group; Data were analyzed by variance analysis using SPSS 16.0 software.

## 4. Discussion

Though Ni is an essential element and its biological actions are not fully understood, it has been proved that its chemical transformations within cells lead to the production of reactive forms of oxygen [27,28]. Some studies have been shown that Ni enhances the oxidation of all DNA bases *in vitro* [29] and lipid peroxidation *in vivo* [30]. The incubation of Ni with cysteine in an aerobic environment generates hydroxyl radicals, which then react with cysteine to generate carbon-centered alkyl radicals. Free radicals can also be generated from lipid hydroperoxides by Ni in the presence of several oligopeptides [31,32]. Misra *et al.* [23] showed that a single intraperitoneal injection of nickel acetate decreased CAT, GSH-Px, GSSG-R activities and GSH concentration, and increased the MDA content, and the activity of SOD were not significantly decreased in rat liver and kidney. However, nickel had no effect on CAT and GSH-Px activities in blood. Sunderman *et al.* [33] also showed that MDA concentrations were significantly increased in the liver and kidney of rats. In agreement with results of the abovementioned studies, our data suggested that dietary NiCl2 caused the intestinal oxidative damage in broilers, which showed a dose and time dependent increase of MDA contents, and decrease of GSH-Px, SOD, CAT activities and GSH contents, ability to inhibit hydroxy radical in the intestines (duodenum, jejunum and ileum). The same results were observed in the serum oxidative stress in broilers fed on diets supplemented with NiCl_2_ in our early studies [34].

The intestinal mucosa is vulnerable to oxidative damage due to the constant exposure to ROS generated by the luminal contents, e.g., oxidized food, transition metals and salivary oxidants [19]. If this oxidative condition proceeds in intestinal cells for an extended period, injuries may occur due to the accumulation of lipid peroxides [35]. It is well known that enhanced ROS generation can overwhelm cell’s intrinsic antioxidant defenses and result in a condition known as “oxidative stress” [36]. Endogenous antioxidants have the capability to prevent the uncontrolled formation of reactive oxygen negative ion. These antioxidants mainly include antioxidant enzymes and non-enzymatic antioxidants [37]. Antioxidant enzymes, such as SOD and CAT, are considered to be the first line of cellular defense against oxidative damage [38]. SOD must work together with antioxidant enzymes, namely GSH-Px and catalase, which remove hydrogen peroxide [39]. In the present study, the activities of antioxidant enzymes including SOD, CAT and GSH-Px in the intestines were all decreased in the 300, 600 and 900 mg/kg groups when compared with those of the control group (Table 1, Table 2, Table 3). The decreased activities of the abovementioned enzymes can lead to an excessive availability of superoxide and hydrogen peroxide in biological systems, which in turn will generate hydroxyl radicals involved in initiation and propagation of lipid peroxidation [40]. In the present study, it was found that the ability to inhibit hydroxy radical in intestines was decreased in the 300, 600 and 900 mg/kg groups when compared with those of control group (Table 4), implying that excess hydroxy radicals accumulated in the intestinal mucosa. Furthermore, hydroxy radical is one of the major oxygen radicals that can cause oxidative stress. Low levels of antioxidant enzyme activities and high levels of free radicals lead to the development of oxidative damage in the intestines. 

Among non-enzymatic antioxidants, glutathione (GSH) plays a primary role and is regarded as an early biological marker of the oxidative stress [41]. In the present study, the GSH content was significantly reduced in the 300, 600 and 900 mg/kg groups from 14 to 42 days of age (Table 5). The reduced glutathione (GSH) was an important cellular antioxidant because of high intracellular concentration and also serves as a substratum of essential scavenger enzymes to maintain oxidative balance [42]. The decreased GSH-Px activity may also be due to the reduced availability of GSH in the present study. 

In addition, the intestinal oxidative damage induced by dietary NiCl_2_ in the present study was also associated with the increased level of lipid peroxidation as measured by MDA production (Table 6). Our data are in agreement with the studies of Sunderman *et al.* [43] that the level of MDA is found to be significantly elevated in serum of NiCl_2_-treated rats. MDA is one of several low-molecular-weight end products that are formed via the decomposition of certain primary and secondary lipid peroxidation products [44]. The MDA production induces alteration of membrane fluidity and increase of membrane fragility [40,45]. Moreover, MDA inhibits various enzyme reactions and exerts mutagenicity and carcinogenicity by forming DNA adducts [46]. High levels of MDA contents imply the enhancement of lipid peroxidation and accumulation of lipid peroxides in the intestinal mucosa, the generation of the reactive species in this condition causes significant the reversible or irreversible oxidative damage to a wide range of biological molecules including DNA, lipids, proteins, carbohydrates or any nearby molecule causing a cascade of chain reactions resulting in intestinal cellular damage. 

## 5. Conclusions

According to the results of the present study and the aforementioned discussion, it is concluded that dietary NiCl_2_ in excess of 300 mg/kg causes inhibition of antioxidant enzyme activities, enhancement of lipid peroxidation and accumulation of free radicals, which consequently induces oxidative damage in the intestinal mucosa of broilers. The intestinal functions including absorptive function and mucosal immune function are finally impaired due to the oxidative damage of the intestinal mucosa. The oxidative damage may be a main mechanism on the effects of NiCl_2_ on the intestinal health. 
